# Genes Associated with Apoptosis in an Experimental Breast Cancer Model

**DOI:** 10.3390/ijms26199735

**Published:** 2025-10-07

**Authors:** Gloria M. Calaf, Leodan A. Crispin

**Affiliations:** Instituto de Alta Investigación, Universidad de Tarapacá, Arica 1000000, Chile; kcrispi@gestion.uta.cl

**Keywords:** apoptosis, breast cancer, *TP63*, *ER*, *p53*

## Abstract

Breast cancer remains a leading cause of global mortality. According to international cancer data, significant progress has been made in treating breast cancer; however, metastasis and drug resistance continue to be the primary causes of mortality for many patients. This study investigated the modulation of apoptosis-related genes in response to ionizing radiation and estrogen exposure based on a human breast epithelial cell model (MCF-10F and its transformed variants: Estrogen, Alpha3, Alpha5, Tumor2) previously established, where cells were treated with high linear energy transfer alpha particles, with or without 17β-estradiol. Gene expression profiling was performed using an Affymetrix U133A microarray, and bioinformatic analyses assessed differential expression, estrogen receptor status, and correlations with overall survival. Distinct gene expression patterns emerged across cell lines and tumor subtypes. *TP53* expression correlated positively with *TP63*, *BIK*, *CFLAR*, *BIRC3*, and *BCLAF1*. *TP63*, *PERP*, *CFLAR*, *BCLAF1*, *GULP1*, and *BIRC3* were elevated in normal tissue, whereas *BIK*, *PHLDA2*, and *BBC3* were upregulated in tumors. ER-positive tumors exhibited higher *TP63*, *BIK*, *BCLAF1*, and *BBC3* expression, while ER-negative tumors showed increased *PERP*, *CFLAR*, *BIRC3*, and *PHLDA2*. Notably, elevated *BCLAF1* expression was associated with poorer survival in Luminal A patients, and high *PHLDA2* expression correlated with reduced survival in Luminal B cases. These findings indicate that resistance to apoptosis is a fundamental mechanism in breast cancer progression and therapeutic evasion. Breast tumors selectively alter the expression of key genes to promote growth, evade apoptosis, and develop therapeutic resistance. The differential expression and correlations of these apoptosis-related genes highlight their potential as molecular targets for future personalized cancer therapies and as valuable biomarkers for prognostic stratification and predicting therapeutic response.

## 1. Introduction

Cancer is a disease of uncontrolled proliferation by transformed cells subject to evolution by natural selection [1]. According to the World Health Organization, among other cancers, breast cancer has emerged as the primary cause of mortality for women globally, accounting for 670,000 fatalities in 2022 [2]. The global increase in the cancer burden, particularly in low-income countries, is attributed to population aging, the adoption of modifiable risk factors (smoking, Western diet, inactivity). Faced with the disproportionate impact of constrained healthcare systems, prevention has become a fundamental strategy [3]. In countries with well-developed healthcare systems, cancer survival rates have increased due to advances in early detection, preventive measures, and access to high-quality treatments [4].

Cancers affecting women, such as breast, uterine, ovarian, endometrial, and cervical cancers, have become increasingly prevalent, and the growing incidence and death rates warrant the development of innovative and alternative approaches to treatments [5]. According to international cancer data, significant progress has been made in treating breast cancer; however, metastasis and drug resistance continue to be the primary cause of mortality for many patients [6]. Breast cancer is a highly heterogeneous disease concerning its molecular, histopathological, and clinical characteristics, including its treatment and prognosis. Then, it is essential to identify the subtype before starting treatment [7]. Patient survival results vary depending on the breast cancer subtype. The molecular subtype of this disease is associated with expression of progesterone receptor (PR), estrogen receptor (ER), and human epidermal growth factor receptor 2 (HER2) [7]. Patients with triple-negative breast cancer (TNBC) do not express the ER receptor, PR, or HER2. TNBC is a subtype that is most aggressive and invasive; it accounts for approximately 15–20% of all breast cancer cases [8]. In comparison with other types of breast cancer, TNBC shows unfavorable prognostic features: increased frequency of visceral metastases, shorter interval without recurrence, and higher nuclear grade [9,10]. The problem of poor prognosis in patients with TNBC results from the limitations of the choice of treatment. Treatment for TNBC consists mainly of doxorubicin, paclitaxel, cyclophosphamide, and immunotherapy [11,12].

There are numerous risk factors known to contribute to developing cancer, such as age, geographic area, and race [2,13]. Other known or suspected risk factors for cancer are alcohol, chronic inflammation, hormones, radiation, sunlight, cancer-causing substances, infectious agents, obesity, tobacco, and others [14]. Altogether, these findings not only deepen our understanding of breast cancer pathophysiology but also open avenues for personalized therapeutic strategies targeting apoptosis resistance. Concerning radiation, radon is a natural radioactive gas produced by the decay of uranium and thorium, furthermore, radon-222 and its decay products are classified as carcinogenic to humans (Group 1) by IARC [15], and it has been associated with lung cancer [16,17,18,19,20,21], leukemia [15,22,23], and basal cell carcinoma [24], among others.

Calaf and Hei established a breast cancer model in 2000 [25], which used human breast cells, MCF-10F [26], to investigate radiation- and estrogen-induced carcinogenesis. The researchers exposed these cells to high linear energy transfer (LET) alpha particles, simulating radon progeny, with and without the presence of 17β-estradiol (estrogen). They observed gradual phenotypic changes in the treated cells, including altered morphology, increased proliferation, anchorage-independent growth, and invasive capability, eventually leading to tumor formation in mice. The study specifically highlights the increased expression of BRCA1, BRCA2, and RAD51 proteins in transformed cells, suggesting that ionizing radiation and estrogen collaborate in the initiation and progression of breast cancer [25]. The present study focused on determining whether genes associated with apoptosis were affected by ionizing radiation and estrogen.

According to the National Cancer Institute, apoptosis is defined as a type of cell death in which molecular processes within a cell lead to its death. This is a method the body uses to get rid of unnecessary or abnormal cells [27]. Commonly called programmed cell death, it plays an essential role in maintaining a balance with mitosis by regulating cell populations during development, preserving tissue homeostasis in adults, and contributing to various biological processes [28].

Apoptosis is a normal and essential process in various contexts. From a physiological perspective, apoptosis is essential during the early stages of development to eliminate unneeded cells, promote cell renewal, and maintain tissue homeostasis. Through this process, the body eliminates aged, defective, or unneeded cells in a controlled manner without inducing inflammation [29]. Among the main risks and complications from a pathological perspective, alterations in apoptotic mechanisms can have serious consequences, such as the inhibition of apoptosis, which is associated with developing cancer, autoimmune diseases, and degenerative diseases, where an excessive activation contributes to conditions such as neurodegenerative diseases, such as Alzheimer’s or Parkinson’s [30]. Apoptosis detection has gained great importance in cancer, as it can be useful for understanding the mechanism of action of drugs, mechanisms associated with the disease, the effectiveness of treatments, etc. Because apoptosis is a dynamic process, related events and their detection depend on multiple factors. Among the processes commonly used to detect apoptosis are the detection of DNA degradation, changes in cell membrane symmetry, and activation of specific proteins [31].

The process of cell death can be induced by intracellular or extracellular signals that determine the need to eliminate a cell in a programmed manner. Mechanistically, there are two main pathways in this process: The intrinsic pathway is activated by internal cellular damage, such as oxidative stress, genotoxic mutations, or the accumulation of misfolded proteins—frequent events in pathologies such as Alzheimer’s and Parkinson’s [32,33]. This process is regulated by the Bcl-2 protein family and the tumor protein p53 (*TP53*) gene, which modulate the release of cytochrome c from the mitochondria, a key event that activates a cascade of executioner caspases, leading to cell death [34,35]. The extrinsic pathway is activated by death signals sent by other cells, which interact with specific receptors such as Fas and TNFR1 on the cell surface [36]. This interaction triggers a cascade that culminates in the activation of caspase-8, initiating the apoptotic process [37,38]. Factors such as the TP53 gene and Bcl-2 proteins regulate the balance between cell survival and death [34,35], while effector caspases, especially caspase-3, carry out the orderly breakdown of the cell [37,38].

Resistance to apoptosis is a key mechanism in breast cancer, where tumor cells evade therapeutic action and perpetuate their growth. Several genes regulate this pathway, playing crucial roles in the balance between cell survival and death [39]. The p53 apoptosis effector related to PMP22 (PERP), the ErbB2/HER2 oncoprotein, is often the triggering factor in breast cancers treated with the anti-ErbB2 antibody trastuzumab. ErbB2 was found to induce breast cancer by inhibiting the expression of the pro-apoptotic protein PERP [40].

CASPASE 9, intrinsic or acquired resistance to chemotherapy, is a major clinical problem that causes death in patients with advanced and metastatic breast cancer. Overexpression of anti-apoptotic genes is thought to influence chemotherapy resistance [41]. Authors [42] found that the Bcl-2 interacting killer (BIK) protein is associated with a poor prognosis for breast cancer, independent of estrogen/progesterone receptor and HER2 status. BIK promotes tumors through a complex mechanism and stimulates autophagy, which contributes to enhanced tumor adaptation. This suggests that BIK-mediated autophagy contributes to disease recurrence.

The BIRC family of genes encodes proteins from the inhibitor of apoptosis (IAP) family, which are crucial for apoptosis resistance in cancers, including breast cancer [43,44]. Among these genes, baculoviral IAP repeat containing 3 (*BIRC3*) stands out for encoding the cIAP1 protein, involved in caspase inhibition and modulation of key signaling pathways such as NF-κB [45,46]. These proteins not only suppress apoptosis but also participate in inflammation, cell proliferation, metastasis, and immune response, regulating signaling and influencing molecular complexes such as the ripoptosome and necrosome, thus evading cell death [47,48,49,50,51].

Researchers identified proteins that interact with PD-L1 and found that the Bcl2-associated transcription factor 1 (*BCLAF1*) is a key regulator, while CMTM6 increases expression, and ATM/BCLAF1/PD-L1 regulates its stability [52]. Pleckstrin homology-like domain family A member 2 (*PHLDA2*) plays an important role in the tumor progression of various cancers, including breast cancer. Its inhibition can promote apoptosis and autophagy in cancer cells, possibly by inactivating the PI3K/AKT/GSK-3β pathways. Its expression is regulated by tumorigenic signals, suggesting a dual role in tumorigenesis and tumor suppression [53,54]. A study revealed that the GULP PTB domain containing engulfment adaptor 1 (*GULP1*) gene can influence cellular resistance to cisplatin-induced apoptosis, increasing cell viability and decreasing apoptosis in cells that overexpress *GULP1*. It has also been identified as a downstream effector of estrogen receptor β, playing a crucial role in apoptotic cell phagocytosis, potentially contributing to cell survival in breast tumors [55,56]. The growth arrest and DNA damage inducible alpha (GADD45) proteins are involved in various cellular processes related to stress signaling and response to cellular injury [57,58,59]. They are rapidly induced in response to oncogenesis, terminal differentiation, and apoptotic cytokines, which can lead to tumor formation [58].

Puma/BBC3 activates mitochondrial apoptosis through direct interaction with Bax and Bak, which are pro-apoptotic members of the Bcl-2 protein family. It plays a critical role in p53-mediated apoptosis, especially in response to cellular stress or DNA damage [60].

Tumor cells can develop resistance to apoptosis through the expression of anti-apoptotic proteins such as Bcl-2 or the reduction in pro-apoptotic proteins such as Bax. Regulation of Bcl-2 and Bax expression appears to be controlled by the tumor suppressor gene p53, which can either suppress or promote mitochondrial membrane changes [61].

There are no specific molecular targets in TNBC to underpin targeted therapy, and one reason for the failure of the applied pharmacotherapy is the inhibition of tumor cell apoptosis [62,63,64,65]. The study of apoptosis has opened new avenues for research in medicine, especially in areas such as oncology and neurodegenerative diseases. Its resistance represents one of the main therapeutic barriers in the treatment of breast cancer. This study aimed to investigate the expression of apoptosis-related genes in an ionizing radiation- and estrogen-induced experimental breast cancer model. Advocating to identify potential biomarkers of resistance to tumors, improve targeted therapies and treatment effectiveness, including a correlation analysis of genes associated with apoptosis, their expression levels in normal and tumor tissues, the relationship between those genes compared to the phenotypic estrogen receptor status (ER status) parameter, and whether the expression of such genes had an impact on the prognosis of breast cancer patients.

## 2. Results

### 2.1. Differential Gene Expression Profile Using an Affymetrix Microarray (U133A) in Breast Cancer

**Figure 1:**
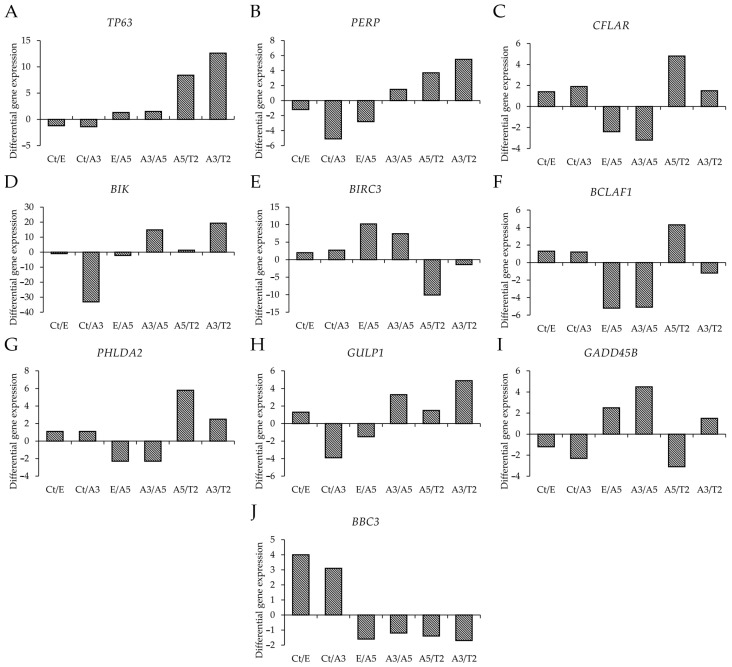
An experimental breast cancer model induced by estrogen and radiation comparing genes such as (**A**) tumor protein p63 (*TP63*), (**B**) p53 apoptosis effector related to PMP22 (*PERP*), (**C**) CASP8 and FADD like apoptosis regulator (*CFLAR*), (**D**) BCL2 interacting killer (BIK), (**E**) baculoviral IAP repeat containing 3 (*BIRC3*), (**F**) BCL2 associated transcription factor 1 (*BCLAF1*), (**G**) pleckstrin homology like domain family A member 2 (*PHLDA2*), (**H**) GULP PTB domain containing engulfment adaptor 1 (*GULP1*), (**I**) growth arrest and DNA damage inducible beta (*GADD45B*), and (**J**) BCL2 binding component 3 (*BBC3*) in cell lines such as MCF-10F/estrogen (Ct/E), Control/Alpha3 (Ct/A3), estrogen/Alpha5 (E/A5), Alpha3/Alpha5 (A3/A5), Alpha5/Tumor2 (A5/T2), and Alpha3/Tumor2 (A3/T2). Graphs were retrieved from a gene expression cluster dendrogram repository from our laboratory for this article.

The selection of genes for this study was based on microarray data derived from a study conducted in 2013 [66], which used the Affymetrix (U133A) microarray commonly employed for transcriptome analysis that enables the quantification of gene expression across various biological contexts, and it is valuable for comparing molecular profiles among cell populations with differing tumor behaviors. Additionally, such a study was based on several cell lines developed by the author in an experimental radiation- and estrogen-induced breast cancer model established in 2000 [25]. Figure 1 compares gene expression levels across different experimental breast cancer cell lines (Ct, A3, A5, and T2).

In Figure 1, it can be observed that *TP63* gene expression was higher in the T2 than in the A3 and A5 cell lines. *PERP* expression was higher in control than in A3, and T2 was higher than in A3 and A5. *CFLAR* gene expression was higher in the E, A3, and T2 cell lines than in the A5 cell line. *BIK* gene expression was higher in Control, A5, and T2 than in A3. *BIRC3* expression was higher in A5 than in E, A3, and T2. *BCLAF1* expression was higher in A5 than in E, A3, and T2. *PHLDA2* gene expression was higher in T2 than in A5. GULP1 expression was higher in control, A5, and T2 than in A3. *GADD45B* gene expression was higher in A5 than in E, A3, and T2. *BBC3* expression was higher in E and A3 than in Control.

### 2.2. Gene Expression Analysis Using Bioinformatics

#### 2.2.1. Correlation Analysis Between TP53 and the Genes Under Study in Breast Cancer Patients

**Figure 2:**
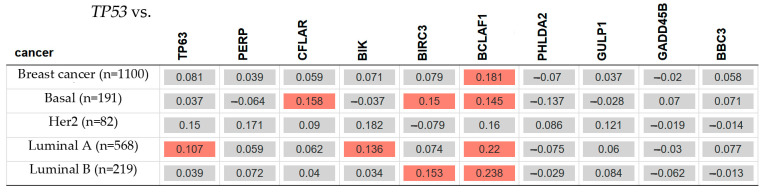
Association between the gene encoding tumor protein p53 (*TP53*) gene expression and a list of genes under study. Heatmap table illustrating the existing correlations between *TP53* and tumor protein p63 (*TP63*), p53 apoptosis effector related to PMP22 (*PERP*), CASP8 and FADD like apoptosis regulator (*CFLAR*), BCL2 interacting killer (*BIK*), baculoviral IAP repeat containing 3 (BIRC3), BCL2 associated transcription factor 1 (*BCLAF1*), pleckstrin homology like domain family A member 2 (*PHLDA2*), GULP PTB domain containing engulfment adaptor 1 (*GULP1*), growth arrest and DNA damage inducible beta (*GADD45B*), and BCL2 binding component 3 (*BBC3*) in breast cancer and subtypes. Red indicates statistically significant positive correlations (Spearman’s, *p* < 0.05), while gray represents non-significant results. The data were collected from TIMER2.0 (http://timer.cistrome.org), accessed on 12 May 2025.

This study employed different datasets from publicly available web servers to investigate the expression levels of genes such as *TP63*, *PERP*, *CFLAR*, *BIK*, *BIRC3*, *BCLAF1*, *PHLDA2*, *GULP1*, *GADD45B*, and *BBC3*. TIMER2.0 [67] was used to explore the association between the tumor protein p53 (*TP53*) gene expression and such genes (Figure 2).

The correlation analysis (Figure 2) between *TP53* gene expression and *TP63*, *PERP*, *CFLAR*, *BIK*, *BIRC3*, *BCLAF1*, *PHLDA2*, *GULP1*, *GADD45B*, and *BBC3* indicated that *TP53* was positively correlated with *TP63* (ρ = 0.107, *p* = 1.06 × 10^−2^) and *BIK* (ρ = 0.136, *p* = 1.2 × 10^−3^) in the Luminal A subtype; with *CFLAR* (ρ = 0.158, *p* = 2.95 × 10^−2^) in the Basal subtype; with *BIRC3* in the Basal and Luminal B subtypes (ρ = 0.15, *p* = 3.82 × 10^−2^ and ρ = 0.153, *p* = 2.33 × 10^−2^, respectively); and with *BCLAF1* in the Basal, the Luminal A and B subtypes (ρ = 0.145, *p* = 4.53 × 10^−2^; ρ = 0.22, *p* = 1.19 × 10^−7^; and ρ = 0.238, *p* = 3.92 × 10^−4^, respectively); however, *PERP*, *PHLDA2*, *GULP1*, *GADD45B*, and *BBC3* were non-significant. Representative scatter plot of the significant results can be seen in the Supplementary Materials Figure S1.

#### 2.2.2. Differential Gene Expression Levels Between Tumor and Normal Tissues in Breast Cancer

**Figure 3:**
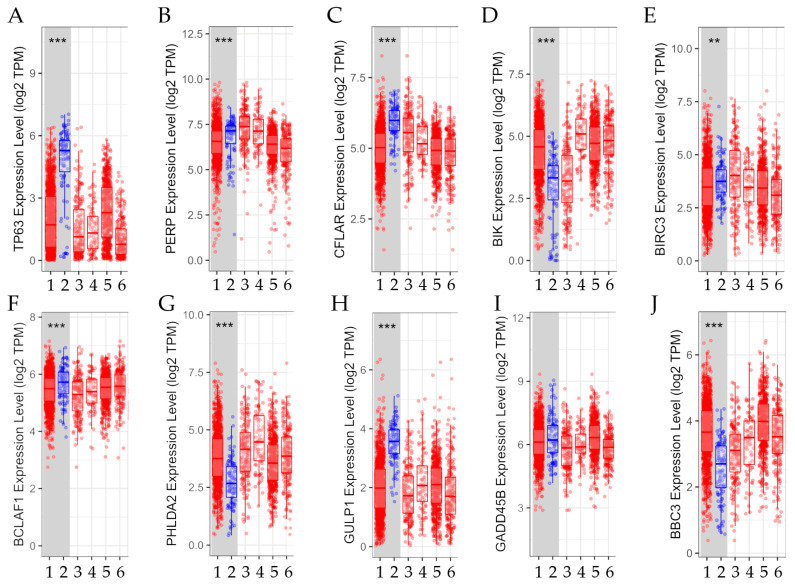
Boxplots illustrate the expression levels of (**A**) tumor protein p63 (TP63), (**B**) p53 apoptosis effector related to PMP22 (PERP), (**C**) CASP8 and FADD like apoptosis regulator (CFLAR), (**D**) BCL2 interacting killer (BIK), (**E**) baculoviral IAP repeat containing 3 (BIRC3), (**F**) BCL2 associated transcription factor 1 (BCLAF1), (**G**) pleckstrin homology like domain family A member 2 (PHLDA2), (**H**) GULP PTB domain containing engulfment adaptor 1 (GULP1), (**I**) growth arrest and DNA damage inducible beta (GADD45B), and (**J**) BCL2 binding component 3 (BBC3) in tumor versus normal tissues (Wilcoxon rank sum test, **: *p*-value < 0.01, ***: *p* < 0.001). Data were extracted from the Tumor Immune Estimation Resource v2.0 (TIMER2.0, http://timer.cistrome.org), accessed on 12 May 2025. Abbreviations: 1: Tumor (n = 1093); 2: Normal (n = 112); 3: Basal Tumor (n = 190); 4: HER2 Tumor (n = 82); 5: Luminal A Tumor (n = 564); 6: Luminal B Tumor (n = 217). Blue denotes normal tissue, and red shows tumors.

Boxplots showing differential gene expression levels (Figure 3).

Results showed that the expression levels of *TP63*, *PERP*, *CFLAR*, *BCLAF1*, and *GULP1* were significantly (*p* < 0.001) higher in normal tissue than in tumor tissue, additionally, *BIRC3* had also a significant (*p* < 0.01) increase in normal tissue in comparison with tumors, whereas *BIK*, *PHLDA2*, and *BBC3* were significantly (*p* < 0.001) higher in tumors than in normal tissues. *GADD45B* expression levels were not significantly different.

#### 2.2.3. Genes Associated with Apoptosis and Estrogen Receptor Status in Breast Cancer Patients

**Figure 4:**
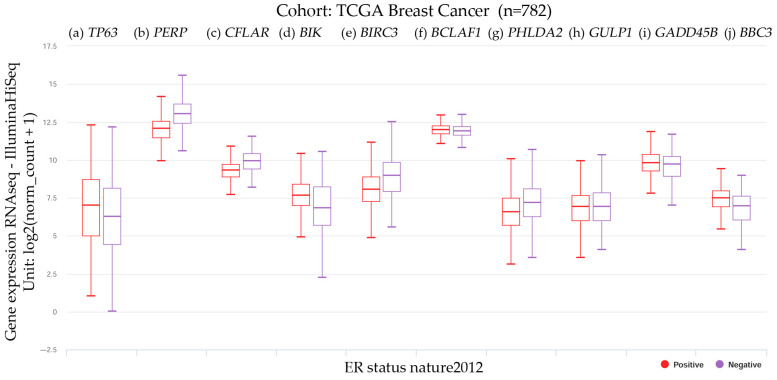
Box plots show gene expression level of (**a**) tumor protein p63 (*TP63*), (**b**) p53 apoptosis effector related to PMP22 (*PERP*), (**c**) CASP8 and FADD like apoptosis regulator (*CFLAR*), (**d**) BCL2 interacting killer (*BIK*), (**e**) baculoviral IAP repeat containing 3 (*BIRC3*), (**f**) BCL2 associated transcription factor 1 (*BCLAF1*), (**g**) pleckstrin homology like domain family A member 2 (*PHLDA2*), (**h**) GULP PTB domain containing engulfment adaptor 1 (*GULP1*), (**i**) growth arrest and DNA damage inducible beta (GADD45B), and (**j**) BCL2 binding component 3 (*BBC3*) in breast cancer. Cohort: TCGA Breast Cancer, n = 782, phenotypically stratified by Nature 2012 for ER status (one-way ANOVA, *p* < 0.05). Data were extracted from the UCSC Xena Functional Genomics Browser (https://xena.ucsc.edu/), accessed on 12 May 2025.

The ER status of *TP63*, *PERP*, *CFLAR*, *BIK*, *BIRC3*, *BCLAF1*, *PHLDA2*, *GULP1*, *GADD45B*, and *BBC3* gene expression levels were studied in breast cancer patients (Figure 4). Such status was analyzed using the UCSC Xena genomic and phenotypic dataset, which included data from the Cancer Genome Atlas (TCGA), International Cancer Genome Consortium (ICGC), and Genomic Data Commons (GDC).

*TP63* (*p* = 0.01647), *BIK* (*p* = 2.216 × 10^−12^), *BCLAF1* (*p* = 0.002227), and *BBC3* (*p* = 1.268 × 10^−10^) expression levels were higher in patients with a positive ER status than those with a negative ER status. On the other hand, *PERP* (*p* = 0.000), *CFLAR* (*p* = 0.000), *BIRC3* (*p* = 1.958 × 10^−10^), and *PHLDA2* (*p* = 9.666 × 10^−7^) were upregulated in patients with negative ER status, whereas *GULP1* and *GADD45B* showed no significant difference.

#### 2.2.4. Survival Evaluation of Breast Cancer Patients

**Figure 5:**
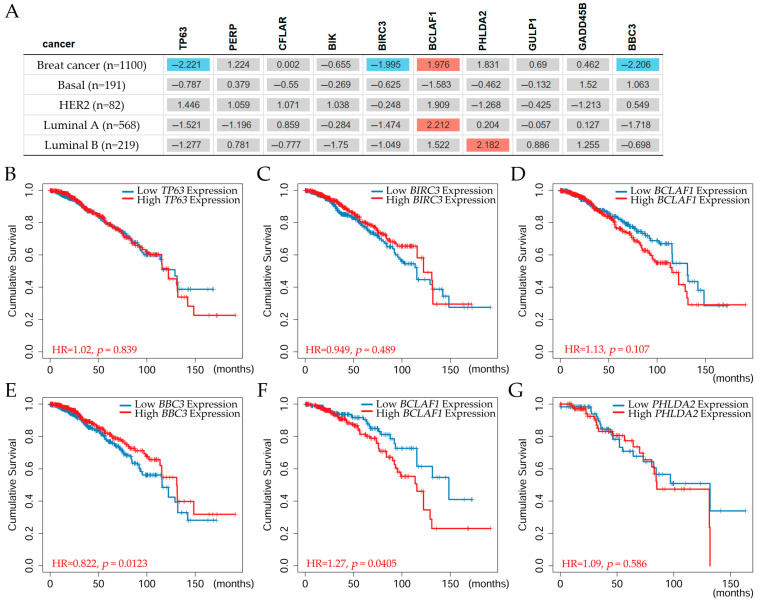
Overall survival analysis of breast cancer patients. (**A**) The heatmap table presents the z-score from TIMER2.0 outcome module evaluating the gene expression of the tumor protein p63 (*TP63*), p53 apoptosis effector related to PMP22 (*PERP*), CASP8 and FADD-like apoptosis regulator (*CFLAR*), BCL2 interacting killer (*BIK*), baculoviral IAP repeat-containing 3 (*BIRC3*), BCL2-associated transcription factor 1 (*BCLAF1*), pleckstrin homology-like domain family A member 2 (*PHLDA2*), GULP PTB domain-containing engulfment adaptor 1 (*GULP1*), growth arrest and DNA damage-inducible beta (*GADD45B*), and BCL2 binding component 3 (*BBC3*) in breast cancer and subtypes, adjusted by the clinical stage factor, The red color indicates a statistically significant increased risk (Z-score, *p* < 0.05), the blue color denotes a statistically significant decreased risk (Z-score, *p* < 0.05), and gray represents a nonsignificant result. The Kaplan–Meier (KM) curve graphs present the cumulative survival and the time to follow-up (months) of the expression levels of (**B**) *TP63*, (**C**) *BIRC3*, (**D**) *BCLAF1*, (**E**) *BBC3* in breast cancer patients; and (**F**) *BCLAF1* in Luminal A and (**G**) *PHLDA2* in Luminal B subtypes. KM survival curves show overall survival based on gene expression levels, grouping patients into high and low expression groups. The information was obtained from TIMER2.0 (http://timer.cistrome.org), accessed on 12 May 2025.

Survival analysis was carried out by TIMER2.0 [67] web server in the context of breast cancer. Within the Gene_Outcome module of TIMER2.0, the impact of gene expression on outcomes was evaluated using the Cox proportional hazard model, which had been adjusted for the clinical stage factor. A heatmap table was generated to illustrate the normalized coefficient of the gene within the Cox model (Figure 5A). By selecting a cell in this heatmap while using the server, the Kaplan–Meier (KM) curves for the genes (Figure 5B–G) were accessed.

In the overall breast cancer cohort (n = 1100), *PERP*, *CFLAR*, *BIK*, *GULP1*, and *GADD45B* gene expression levels were non-significant (Figure 5A); however, *TP63*, *BIRC3*, and *BBC3* expression levels were significantly (z-score: *p* < 0.05, z > 0) associated with a decreased risk of breast cancer. *BCLAF1* expression was significantly (z-score: *p* < 0.05, z > 0) associated with an increased risk of breast cancer, indicating that higher expression of *BCLAF1* was linked to poorer overall survival in breast cancer patients. Conversely, higher expression of *TP63*, *BIRC3*, and *BBC3* was associated with improved overall survival in the general breast cancer population. Notably, while *TP63*, *BIRC3*, and *BBC3* showed an effect on overall survival, this effect did not appear to differ significantly across breast cancer subtypes. For patients specifically within the Luminal A subtype (n = 568), *BCLAF1* gene expression levels showed a significantly (*p* < 0.05) increased risk of breast cancer. In the Luminal B subtype (n = 219), *PHLDA2* gene expression levels also depicted a significantly (*p* < 0.05) increased risk of this disease.

Kaplan–Meier (KM) analysis revealed that patients with high *TP63* (Figure 5B), *BIRC3* (Figure 5C), and *BBC3* (Figure 5E) expression levels were significantly associated with overall survival, with a Hazard Ratio (HR) of 1.02 (*p* = 0.839); 0.949 (*p* = 0.489); and 0.822 (*p* = 0.0123), respectively. However, high *BCLAF1* (Figure 5D) expression experienced a substantial decrease in cumulative survival of about 70% at approximately 135 months with a HR of 1.13 (*p* = 0.107). Additionally, high *BCLAF1* (Figure 5F) expression showed a decrease in cumulative survival of about 80% at approximately 135 months. The Hazard Ratio (HR) for *BCLAF1* in Luminal A was 1.27 (*p* = 0.0405). This further emphasized that elevated *BCLAF1* levels were detrimental to survival in this specific subtype. KM analysis showed that patients with high *PHLDA2* expression in the Luminal B subtype did not survive beyond 140 months (Figure 5G). The Hazard Ratio (HR) for *PHLDA2* was 1.09 (*p* = 0.586), which indicated that high *PHLDA2* expression was associated with a significantly poorer prognosis in Luminal B breast cancer.

## 3. Discussion

Disrupting the apoptotic process has been identified as one hallmark of cancer, allowing genetically damaged cells to evade physiological elimination and contribute to tumor progression [68]. This process can be blocked in cancer cells, playing a key role in maintaining the balance with mitosis by regulating cell populations during development, preserving tissue homeostasis, and contributing to various biological processes [28].

Abnormalities in regulating cell death may be a critical factor in diseases such as cancer. Some conditions are characterized by insufficient apoptosis, while others show excessive apoptosis [69]. Cancer represents a breakdown in the normal regulatory mechanisms of the cell cycle, manifesting as excessive cell proliferation or reduced cell elimination [70]. The inhibition of apoptosis during carcinogenesis is considered to play a crucial role in the development and progression of certain cancer types [71]. Tumor cells can acquire resistance to apoptosis through the expression of anti-apoptotic proteins such as Bcl-2 or the regulation or mutation of pro-apoptotic proteins like Bax. The activity of Bcl-2 and Bax is controlled by the tumor suppressor gene p53 [61].

*TP63* expression was higher in the T2 cell line than in A3 and A5, suggesting the activation of p53-mediated apoptotic mechanisms [72]. *PERP* expression was higher in the control group than in A3, and also higher in T2 compared to A3 and A5, indicating these latter lines exhibited deregulation of the p53-dependent apoptotic pathway. Its reduction in A3 and A5 may reflect pathway inactivation and increased tumor aggressiveness [73]. *CFLAR* was more highly expressed in T2 than in A5. Since this gene inhibits extrinsic apoptosis via caspase-8, its reduced expression in A5 may indicate dysfunction in apoptotic regulatory mechanisms [74]. *BIK* expression was higher in C, A5, and T2 compared to A3, suggesting an active pro-apoptotic profile that may confer greater sensitivity to cellular damage [75]. *BIRC3* showed increased expression in A5, suggesting the use of apoptosis-inhibiting mechanisms associated with therapeutic resistance [76]. *BCLAF1* expression was higher in E, A3, and T2, and is involved in DNA repair and transcriptional control. Its reduced expression in A5 may reflect a loss of genomic regulation [77]. *PHLDA2* was overexpressed in T2, possibly linked to compensatory pathways or negative regulation of the PI3K/AKT axis [53]. *GULP1* expression was higher in C, A5, and T2, suggesting enhanced apoptotic cell clearance mechanisms, potentially in response to increased cell death [55]. *GADD45B*, more highly expressed in A5 than in the other groups, suggests adaptation to genotoxic stress or alterations in cell cycle signaling [78]. Finally, *BBC3* (PUMA) was overexpressed in T2, supporting the activation of the mitochondrial apoptotic pathway, directly regulated by p53 [79].

Data obtained from TIMER2.0 revealed statistically significant positive correlations. Panel (A) displays Spearman correlation coefficients between *TP53* expression and several apoptosis-related genes, including *TP63*, *PERP*, *CFLAR*, *BIK*, *BIRC3*, *BCLAF1*, *PHLDA2*, *GULP1*, *GADD45B*, and *BBC3*. Red-colored cells represent statistically significant positive correlations, whereas gray cells indicate non-significant results. This visualization facilitates the detection of co-expression patterns between *TP53* and other genes in breast cancer, highlighting those with consistent correlations across various tumor subtypes. Panel (B) shows individual boxplots for the genes that demonstrated the strongest and most consistent correlations with *TP53*, such as *TP63*, *CFLAR*, *BIK*, *BIRC3*, and *BCLAF1*. These genes were selected for graphical representation based on the magnitude and robustness of their correlation with *TP53*, illustrating how their expression is linked to that of *TP53* across molecular breast cancer subtypes. With *TP63*, the positive correlation suggests that in contexts where *TP53* is active, *TP63* may also be upregulated, potentially enhancing the response to genomic damage. It regulates epithelial maintenance and may contribute to apoptosis or tumor aggressiveness, depending on the predominant isoform [80]. The positive correlation with *CFLAR* supports findings that identify it as a *TP53* transcriptional target [81]; *CFLAR* can inhibit the extrinsic apoptosis pathway by blocking caspase-8, preventing death receptor-induced apoptosis [74]. *BIK*, a pro-apoptotic mitochondrial effector, shows a positive correlation, consistent with its role as a direct p53 target in response to stress signals [75]. This suggests that in certain tumors, *TP53* retains its canonical pro-apoptotic function. *BIRC3* is a component of the NF-κB pathway, and its correlation may reflect an adaptive resistance mechanism in tumors with sustained *TP53* activity, helping to balance proliferation and cell death [82]. Its positive correlation with *TP53* may also indicate an attempt by the tumor to modulate cell death or immune responses [76]. *BCLAF1* is involved in DNA repair and cell cycle regulation. In the presence of moderate damage, *TP53* may activate *BCLAF1* to delay cell death and preserve genomic stability. Its positive correlation may reflect a conservative *TP53* response aimed at initiating repair over immediate apoptosis [77]. The graphical representation suggests a consistent positive association between *TP53* expression and several apoptosis-related genes, indicating a potential coordinated response to cellular stress. This supports the concept of *TP53* as a central regulatory hub that integrates damage signals, apoptosis, and survival responses, depending on the molecular context of the tumor. The bioinformatic validation of these correlations underscores their value as a foundation for identifying potential biomarkers or therapeutic targets in breast cancer.

Significant differences in gene expression were observed between tumor and normal breast tissue, based on data retrieved from TIMER 2.0, enabling reliable comparative transcriptomic profiling. Most genes evaluated showed reduced expression in tumor tissue compared to normal tissue, including *TP63*, *PERP*, *CFLAR*, *BIRC3*, *BCLAF1*, *GULP1*, and *GADD45B*. *TP63*, whose ΔNp63 isoform has been linked to cell proliferation and resistance to apoptosis, displayed lower expression in tumor tissue. This finding may indicate transcriptional suppression of *TP63* in specific breast cancer contexts, contrasting with its reported oncogenic role under other conditions [72]. With *PERP*, reduced expression has been associated with increased tumor aggressiveness, apoptosis resistance, and higher recurrence risk in breast cancer [73]. *CFLAR*, a blocker of caspase-8 activation that interferes with the extrinsic apoptosis pathway, may be downregulated in tumor tissue due to cellular reprogramming favoring alternative resistance mechanisms [74]. *BIRC3*, a member of the inhibitor of apoptosis (IAP) family, exhibits both pro- and anti-apoptotic functions depending on the cellular context. Its reduced expression in tumors may reflect dysregulation within the NF-κB axis or shifts in tumor signaling requirements [76]. *BCLAF1*, associated with pro-apoptotic activity and DNA repair, may also promote cell survival under stress conditions. Its downregulation could impair genome maintenance and promote instability, facilitating tumor growth [77]. *GULP1*, involved in apoptotic cell clearance and hormonal signaling, may be under-expressed as a mechanism of immune evasion or impaired apoptotic clearance [55]. *GADD45B*, which regulates the cell cycle in response to genotoxic stress, is also downregulated, suggesting loss of damage control and tumor adaptation to stress conditions [78]. *PHLDA2*, *BBC3*, and *BIK* were overexpressed in tumor tissue. *PHLDA2*, a negative regulator of the PI3K/AKT pathway, has been associated with tumor progression, and its overexpression may reflect compensatory mechanisms or epigenetic alterations within the tumor microenvironment [53]. *BBC3*, a pro-apoptotic p53-regulated gene, may be upregulated in response to mitochondrial damage or to compensate for inhibition of other apoptotic pathways [79]. Last, *BIK*, which plays a complex role in hormone-related tumors, may indicate persistent pro-apoptotic signaling in certain subtypes when highly expressed [75]. These findings demonstrate that breast tumors can selectively alter the expression of key genes to promote growth, evade apoptosis, and develop therapeutic resistance—highlighting potential molecular targets for future personalized cancer therapies.

The association with estrogen receptor (ER) status, based on transcriptomic data analysis from the UCSC Xena Functional Genomics Browser, reveals that certain genes exhibit patterns of differential expression. Among the genes with higher expression in ER-positive (ER^+^) breast tumors are *TP63*, *BIK*, *BCLAF1*, *GADD45B*, and *BBC3*. *TP63*, although traditionally associated with poorly differentiated tumors, may also express active isoforms in ER^+^ tumors, performing pro-apoptotic or epithelial differentiation functions [72]. BIK, a mitochondrial pro-apoptotic gene, shows elevated expression associated with enhanced p53 functionality in hormone-dependent tumors [75]. *BCLAF1*, involved in DNA repair and immune regulation, may act as a positive modulator in hormone-sensitive contexts [77]. *GADD45B*, which participates in cell cycle arrest and DNA damage response, exhibits higher expression in ER^+^ tumors, potentially indicating increased genomic stability [78]. *BBC3*, a p53-regulated pro-apoptotic factor, is also more highly expressed in contexts where p53 remains wild-type, a condition more frequently observed in ER^+^ tumors [79]. Conversely, genes with higher expression in ER-negative (ER^−^) tumors include *PHLDA2*, *CFLAR*, *BIRC3*, *PERP*, and *GULP1*. *PHLDA2*, involved in the PI3K/AKT signaling pathway, is overexpressed in ER^−^ tumors, suggesting proliferative dysregulation [53]. *CFLAR* and *BIRC3*, both inhibitors of apoptosis, show increased expression in ER^−^ tumors, likely representing mechanisms of apoptotic resistance [74,76]. *PERP*, a p53 effector involved in pro-apoptotic signaling, displays reduced expression in ER^−^ tumors, potentially reflecting impaired regulation due to TP53 mutations [73]. Last, *GULP1*, associated with apoptotic cell clearance and hormonal regulation, shows elevated expression in ER^−^ tumors, possibly as an adaptive mechanism to the tumor microenvironment [55].

**Scheme 1:**
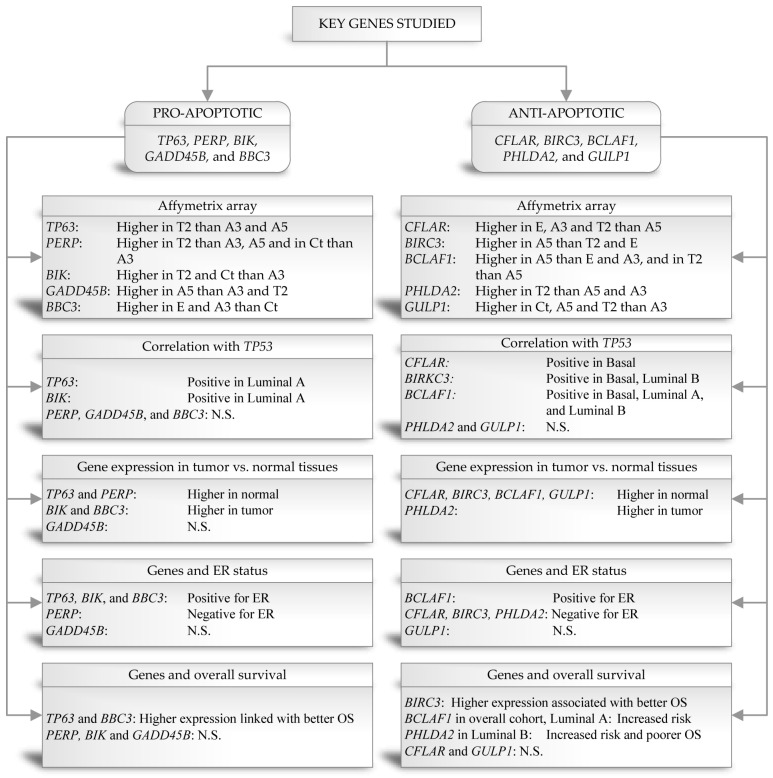
Genes associated with apoptosis in an experimental breast cancer model. Summary of the main findings observed in this work. Key genes are divided into pro-apoptotic ones, such as *TP63*, *PERP*, *BIK*, *GADD45B*, and *BBC3*, and anti-apoptotic ones such as *CFLAR*, *BIRC3*, *BCLAF1*, *PHLDA2*, and *GULP1*. Abbreviations. N.S.: non-significant, OS: Overall survival.

In the overall survival analysis adjusted for clinical stage, data obtained from the TIMER2.0 resource highlighted differential survival associations based on the expression levels of individual genes, providing crucial insights for prognostic stratification and therapeutic potential. High expression of *BCLAF1* was associated with poorer overall survival, suggesting its potential role as a negative prognostic marker. This gene was linked to apoptosis resistance and chemoresistance, likely due to its interference with p53-mediated signaling pathways [83]. *TP63* and *PERP* exhibit dual roles depending on tumor subtype, acting as tumor suppressors or growth promoters depending on the cellular microenvironment [84,85]. Genes such as *CFLAR*, *BIRC3*, and *BIK* participated in both pro- and anti-apoptotic pathways, but their prognostic impact might be modulated by compensatory regulators or restricted to specific cellular activation states [86,87,88]. Last, genes like *GULP1*, *GADD45B*, and *BBC3*, although involved in DNA damage response, might not serve as sole determinants of clinical prognosis due to their redundant regulation or subtype-specific roles [89,90,91]. The following Scheme 1 summarizes the key genes related to pro-apoptotic and anti-apoptotic genes, including the Affymetrix array analysis, correlations with TP53, gene expression in tumor and normal tissues, the ER status, and overall survival.

In summary, this study investigated how ionizing radiation and estrogen influence the expression of apoptosis-related genes in breast cancer, using a well-established MCF-10F cell model. Experimental Design consisted of an established model with the human breast epithelial MCF-10F cell line and transformed variants (Estrogen, Alpha3, Alpha5, Tumor2). The treatment consisted of High linear energy transfer (LET) alpha particles ± 17β-estradiol. The analysis was performed with an Affymetrix U133A microarray and bioinformatics (TIMER2.0, UCSC Xena).

**Scheme 2:**
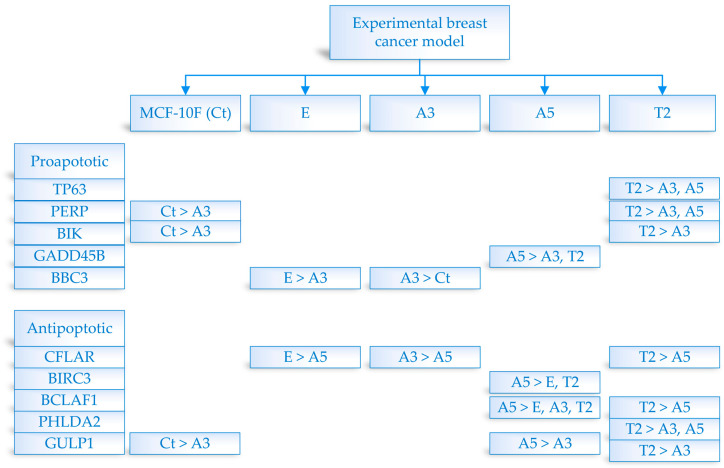
Genes associated with pro- and anti-apoptotic genes in an experimental breast cancer model. Genes were consistently upregulated in the MCF-10F progression model. The connection between the experimental microarray data and the public dataset analyses indicated such progression. This scheme highlights the results along the progression. A summary of the main findings is presented in this work. Key genes are divided into pro-apoptotic ones, such as *TP63*, *PERP*, *BIK*, *GADD45B*, and *BBC3*, and anti-apoptotic ones such as *CFLAR*, *BIRC3*, *BCLAF1*, *PHLDA2*, and *GULP1*.

The key genes studied were pro-apoptotic, such as *TP63*, *PERP*, *BIK*, *BBC3*, and *GADD45B*, and anti-apoptotic, such as *CFLAR*, *BIRC3*, *BCLAF1*, *PHLDA2*, and *GULP1*. Among the pro-apoptotic genes, a positive correlation was observed between *TP53* versus *TP63* and *BIK* in the Luminal A subtype; however, there was no significance with PERP, GADD45B, and BBC3. Among the anti-apoptotic genes, there was a positive correlation between TP53 and *CFLAR*,* BIRC3*, and *BCLAF1* in the Basal subtype; between TP53 and *BIRC3* in Luminal B subtypes; and between TP53 and *BCLAF1* in the Luminal A and B subtypes. However, there was no significant difference between TP53 and *PHLDA2*, *GULP1*. The major findings in relation to normal and tumor tissue indicated that *TP63*, *PERP*, *CFLAR*, *BCLAF1*, *GULP1*, and *BIRC3* gene expression levels were higher in normal than malignant tissues. However, *BIK*, *PHLDA2*, and *BBC3* were higher in tumor tissues. There was no significant difference in *GADD45B*. In relation to ER status, it was found that *TP63*, *BIK*, *BCLAF1*, and *BBC3* were present in ER-positive tumors, whereas *PERP*, *CFLAR*, *BIRC3*, and *PHLDA2* were present in ER-negative tumors. There was no significant difference in ER status when *GULP1* and *GADD45B* were analyzed. The survival analysis showed that high *BCLAF1* expression had poor survival in Luminal A, whereas high *PHLDA2* expression indicated poor survival in Luminal B breast cancer patients. However, high *TP63*, *BIRC3*, and *BBC3* expressions had better overall survival. Genes present in the cell lines of the model (Scheme 2).

The genes consistently upregulated in the MCF-10F progression model and in TCGA breast cancer samples were analyzed. In relation to *TP53*, among the genes upregulated in T2 were *TP63*, *PERP*, *BIK*, *CFLAR*, *BCLAF1*, *PHLDA2*, and *GULP1* in the model, whereas *TP63*, *BIK*, *CFLAR*, *BIRC3*, and *BCLAF1* were positive in breast cancer patients. Concerning tumor versus normal tissues, *PERP*, *BIK*, and *GULP1* were found in normal tissues and *TP63*, *PERP*, *BIK*, *CFLAR*, *BCLAF1*, *PHLDA2*, and *GULP1* were found in T2 in the model, whereas *TP63*, *PERP*, *CFLAR*, *BIRC3*, *BCLAF1*, and *GULP1* were found in normal tissues of patients and *BIK* and *BBC3*, and *PHLDA2* were found in breast cancer tissues.

The genes found in the cell line were *TP63* in T2, *PERP* and *BIK* in Ct and T2, *GADD45B* in A5, *BBC3* in E and A3, *CFLAR* in E, A5, and T2, *BIRC3* in A5, *BCLAF1* in A5 and T2, *PHLDA2* in T2, and *GULP1* in Ct, A5, and T2 cell line. The patients positive for ER had *TP63*, *BIK*, *BBC3*, and *BCLAF1* gene expression, and those with negative ER had *PERP*, *CFLAR*, *BIRC3*, and *PHLDA2*. *BLCAF1* had the worst survival among the genes, which was also present in the A5, a pre-tumorigenic, and T2, the tumorigenic cell line. It can be concluded that breast tumors selectively modulate apoptotic gene expression to evade cell death and resist therapy; *BCLAF1* and *PHLDA2* emerge as potential negative prognostic markers, and *TP63* and *BBC3* may serve as protective biomarkers. These findings support the development of personalized therapies targeting apoptotic pathways.

## 4. Materials and Methods

### 4.1. The Experimental Model

Calaf and Hei’s experimental breast cancer model was established in 2000 [25]. Such a study utilized the spontaneously immortalized human breast epithelial cell line, MCF-10F (ATTC), as its starting material. The research focused on generating transformed variants of the MCF-10F cells through exposure to radiation and estrogen to establish an experimental breast cancer model. The cell lines “produced” in that study were essentially the MCF-10F variants that underwent specific treatments and exhibited altered characteristics indicative of neoplastic transformation. These variants were identified based on their exposure to high linear energy transfer (LET) α particles and/or the presence of 17β-estradiol (E) during culture. The α particles (150 keV/μm) were accelerated to 4 MeV using the van de Graaff accelerator at the Radiological Research Facilities of Columbia University. The key cell line that represents the successful establishment of a radiation- and estrogen-induced breast cancer model is the MCF-10F 60E/60E variant. This cell line, specifically referred to as the “60 cGy+E/60 cGy+E α particle-treated cell line,” was the only one that consistently produced tumors when injected into nude mice, demonstrating tumorigenicity. It also showed anchorage-independent growth and increased expression of BRCA1, BRCA2, and RAD51 proteins. Other transformed MCF-10F variants produced and characterized in the study, which showed various degrees of phenotypic changes (like altered morphology, increased cell proliferation, anchorage-independent growth, and invasive capability, but not always tumorigenicity), included those exposed to single or double doses of 30, 60, or 100 cGy of α particles (^4^He ions). These radiation treatments were applied in the presence or absence of 17β-estradiol (E) at 1 × 10^−8^ mol/L. Some of the produced variants included the MCF-10F 60 × 1 (single 60 cGy α particle dose), the MCF-10F + E 60 × 1 (single 60 cGy α particle dose with estrogen), the MCF-10F 60 × 2 (double 60 cGy α particle dose), the MCF-10F + E 60 × 2 (double 60 cGy α particle dose with estrogen), and the MCF-10F 60E/60 (60 cGy α particle dose cultured with estrogen, followed by another 60 cGy dose), among others. The creation of these specific variants of MCF-10F cells, particularly the tumorigenic 60 cGy+E/60 cGy+E α particle-treated cell line (MCF-10F 60E/60E), was central to the aim of the study, which was to understand breast cancer pathogenesis and the mechanisms of neoplastic transformation induced by high LET radiation in the presence of estrogen.

### 4.2. Cell Lines for Microarray Analysis

The experimental breast cancer model established by Calaf and Hei (2000) [25] provided, among others, five distinct human breast epithelial cell lines (variants of MCF-10F cells). The MCF-10F cells, obtained from ATCC, were cultured in a DMEM/F-12 (1:1) medium enriched with antibiotics [100 U/mL penicillin, 100 μg/mL streptomycin, 2.5 μg/mL amphotericin B (all procured from Life Technologies, Grand Island, NY, USA)] along with 10 μg/mL and 5% equine serum (Biofluids, Rockville, MD, USA). Additionally, the medium included 0.5 μg/mL hydrocortisone (Sigma, St. Louis, MO, USA) and 0.02 μg/mL epidermal growth factor (Collaborative Research, Bedford, MA, USA) [25,26,92,93,94,95,96]. Each cell line representing different stages of transformation such as (i) the MCF-10F (Ct), the parental or control cell line, an immortalized human breast epithelial cell line, did not exhibit features of malignant cells such as anchorage-independent growth, invasion, or tumor growth in nude mice; (ii) the Estrogen (E) cell line, MCF-10F cells that were continuously grown with 17β-estradiol at 10^−8^ M. Like the parental MCF-10F, it also did not display malignant characteristics; (iii) the Alpha3 (A3), this cell line was derived from MCF-10F cells exposed to low doses of high linear energy transfer (LET) α particle radiation. It is described as a non-malignant cell line, but it showed some transformed properties by forming colonies in soft agar and invading, though it failed to form tumors in immunosuppressed mice; (iv) the Alpha5 (A5), this cell line was also developed from MCF-10F through exposure to high LET α particle radiation and subsequent growth in the presence of estrogen, leading to a malignant and tumorigenic phenotype. The A5 cell line induced mammary gland tumors in animals after cell injection, and (v) the Tumor2 (T2) cell line was derived from cells originating from a tumor that formed after injecting Alpha5 cells into nude mice.

### 4.3. Microarray Gene Expression Analysis

Calaf et al. (2013) [66] employed a gene microarray analysis using several MCF-10F variants mentioned above to determine whether genes were affected by estrogen alone, ionizing radiation, or both combined. The gene expression analysis using microarrays involved several key steps, as described in Calaf et al. (2013) [66], such as mRNA isolation, fluorescence-labeled probe preparation, hybridization, microarray platform where the study specifically used the Affymetrix HG-U133A Plus 2.0 GeneChip microarray (Affymetrix, Santa Clara, CA, USA), this microarray contains probes for approximately 14,500 genes; and a Quantitative Analysis where gene expression was quantitatively analyzed using the Affymetrix GeneChip Operating software (GCOS) v1.0 ST. A dual global scaling option within the Genes@Work v1.0 software platform, utilizing the discovery algorithm SPLASH (structural pattern localization analysis by sequential histograms), was applied. A false discovery rate of 0.05 was used for this analysis.

The study presented fold-change and pairwise analysis of differentially expressed genes in a breast cancer model, including (i) the comparison Ct/E that was conducted to evaluate the impact of estrogen alone against a control group, focusing on the effects of estrogen; (ii) Ct/A3 was utilized to assess the impact of ionizing radiation alone compared to the control, with a focus on radiation effects; (iii) E/A5 examined the influence of estrogen in conjunction with ionizing radiation, aiming to evaluate the radiation effect; (iv) A3/A5 compared the effects of radiation alone and combined with estrogen, to study their joint impact; (v) A5/T2 analyzed the effects of both estrogen and ionizing radiation against environmental factors in athymic animals; and (vi) A3/T2 investigated the influence of the microenvironment and radiation alone.

### 4.4. Bioinformatics and Statistical Analysis

This article utilized data from web-based platforms such as the Tumor Immune Estimation Resource v2.0 (TIMER2.0) available at http://timer.cistrome.org, accessed on 1 August 2024 [67], and the University of California, Santa Cruz (UCSC) Xena functional genomics explorer, available at https://xena.ucsc.edu/, accessed on 1 August 2024 [97]. These datasets were employed to investigate the mRNA expressions of *TP63*, *PERP*, *CFLAR*, *BIK*, *BIRC3*, *BCLAF1*, *PHLDA2*, *GULP1*, *GADD45B*, and *BBC3* in breast cancer. This investigation also incorporated data from The Cancer Genome Atlas (TCGA) and The Genotype-Tissue Expression (GTEx) projects. Correlation analysis was performed through the Gene-Corr module of TIMER2.0 of the Exploration component, with significance determined by Spearman’s test. The GeneDE module within the Cancer Exploration component of TIMER2.0 facilitated the analysis of tumor versus normal tissues, with statistical significance evaluated via the Wilcoxon test, denoted by asterisks (*: *p*-value < 0.05; **: *p*-value < 0.01; ***: *p*-value < 0.001). The UCSC Xena web server (https://xena.ucsc.edu/, accessed on 1 August 2024) provided an online visualization of the estrogen receptor status of the genes in question, and statistical significance was measured using a one-way ANOVA test. Overall survival (OS) analysis was executed using the Gene_Outcome module of TIMER2.0, which applied the Cox proportional hazard model, modified by the clinical stage factor, to determine the significance of outcomes. A heatmap table was generated to display the normalized coefficient of the gene in the Cox model. Kaplan–Meier (KM) curves of the genes were accessible by selecting a cell within the heatmap on the web server. The statistical significance of the KM survival curves was evaluated using the log-rank test, with *p* < 0.05 being indicative of a statistically significant difference.

## 5. Conclusions

Apoptosis, a critical mechanism for maintaining cellular homeostasis, is frequently disrupted in breast cancer, contributing to tumor progression and resistance to therapy. This study reveals that breast cancer cells selectively modulate the expression of apoptosis-related genes—such as *TP53*, *TP63*, *BIK*, *CFLAR*, *BIRC3*, and *BCLAF1*—in response to ionizing radiation and estrogen. These alterations vary by tumor subtype and estrogen receptor status, with distinct gene expression patterns linked to poorer survival outcomes in Luminal A and B cancers. The findings underscore apoptosis evasion as a central strategy in breast cancer aggressiveness and highlight key genes as potential biomarkers and therapeutic targets for overcoming resistance and improving patient outcomes. *BCLAF1* and *PHLDA2* are associated with a significantly increased risk and poorer survival, especially within specific breast cancer subtypes (Luminal A and Luminal B, respectively). Conversely, higher expression of BBC3 and TP63 appears to be associated with better overall survival in general breast cancer patients.

## Data Availability

The data in this study are openly available in the Tumor Immune Estimation Resource v2.0 (TIMER2.0) [67], freely available at http://timer.cistrome.org (accessed on 1 August 2024); the University of California, Santa Cruz, UCSC Xena functional genomics explorer [97], freely available at https://xena.ucsc.edu/, accessed on 1 August 2024. The data generated in the present study may be requested from the corresponding author.

## Supplementary Materials

The following supporting information can be downloaded at: https://www.mdpi.com/article/10.3390/ijms26199735/s1.

## Abbreviations

These abbreviations are used in this manuscript:

TLA	Three-letter acronym
PR	Progesterone receptor
ER	Estrogen receptor
HER2	Human epidermal growth factor receptor 2
TNBC	Triple-negative breast cancer
LET	Linear energy transfer
PERP	p53 apoptosis effector related to PMP22
BIK	Bcl-2-interacting killer
BIRC3	Baculoviral IAP repeat containing 3
BCLAF1	Bcl2-associated transcription factor 1
PHLDA2	Pleckstrin homology-like domain family A member 2
GULP1	GULP PTB domain-containing engulfment adaptor 1
GADD45B	Growth arrest and DNA damage-inducible beta
TP63	Tumor protein p63
BBC3	BCL2 binding component 3
TP53	Tumor protein p53
TCGA	The Cancer Genome Atlas
ICGC	International Cancer Genome Consortium
GDC	Genomic Data Commons
