# Supplementary material for: Genes Associated with Apoptosis in an Experimental Breast Cancer Model
Source: Int J Mol Sci. 2025 Oct 7;26(19):9735. doi: 10.3390/ijms26199735 (PMC12524443; doi:10.3390/ijms26199735)
Supplement: Supplementary file 1 [file ijms-26-09735-s001.zip › ijms-3887459-supplementary.pdf]

Significant correlations between *TP53* expression and *TP63, CFLAR, BIK, BIRC3,* and *BCLAF1* expression levels in breast cancer subtypes

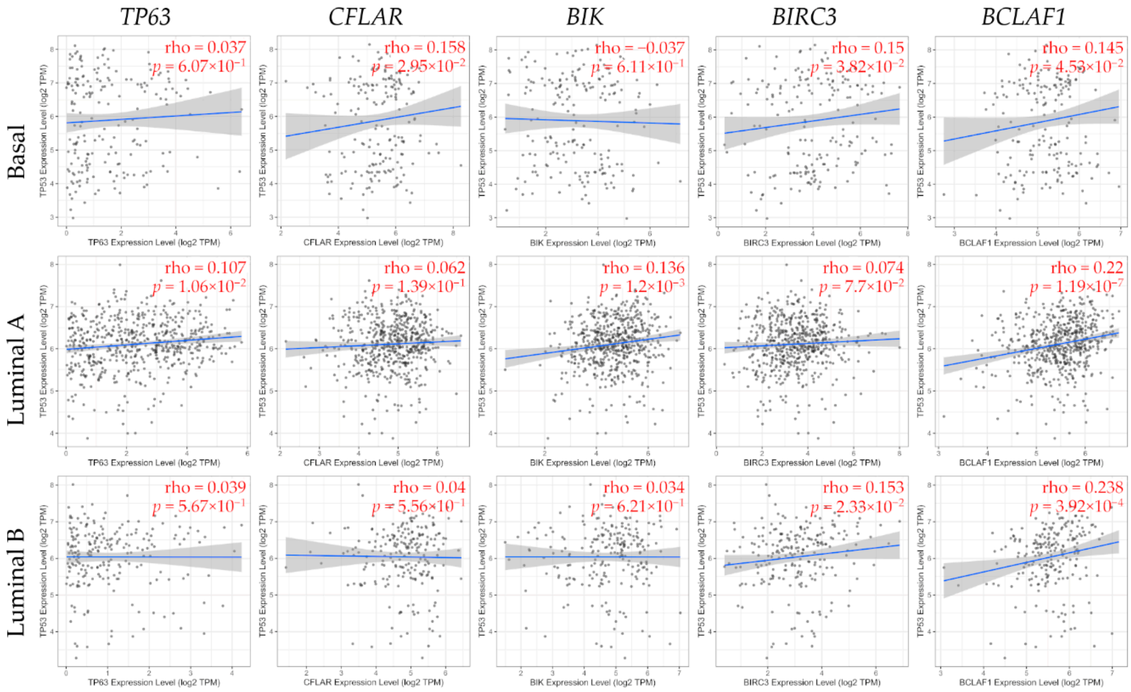

Figure S1. The scatter plots showing significant correlations between *TP53* expression and the expression levels of *TP63*, *CFLAR*, *BIK*, *BIRC3*, and *BCLAF1* across breast cancer subtypes. The scatter plots presented include linear regression lines and correlation coefficients (ρ). In each plot, the blue lines illustrate the linear regression fit, highlighting the trend or connection between the expression levels of *TP53* and the corresponding gene. Surrounding each blue regression line, the gray-shaded area represents the confidence interval. Correlation values for each analysis are shown in red on the right (Spearman's, $p < 0.05$). The data retrieved from TIMER2.0 (http://timer.cistrome.org), accessed on May 12, 2025.
